# Psychometric evaluation of separate anxiety and depression item banks - potential supplements to the EORTC CAT Core

**DOI:** 10.1007/s11136-026-04370-1

**Published:** 2026-08-12

**Authors:** Morten Aa. Petersen, Hugo Vachon, Neil K. Aaronson, Johannes M. Giesinger, Bernhard Holzner, Monika Sztankay, Mogens Groenvold

**Affiliations:** 1 Palliative Care Research Unit, Department of Geriatrics and Palliative Medicine, Copenhagen University Hospital, Bispebjerg & Frederiksberg, Bispebjerg bakke 23B, 2400 Copenhagen NV, Denmark; 2 Quality of Life Department, European Organization for Research and Treatment of Cancer, Brussels, Belgium; 3 Division of Psychosocial Research and Epidemiology, Netherlands Cancer Institute, Amsterdam, The Netherlands; 4 Health Outcomes Research Unit, University Hospital of Psychiatry II, Innsbruck Medical University, Innsbruck, Austria; 5 Department of Public Health, University of Copenhagen, Copenhagen, Denmark

**Keywords:** Anxiety, Computerized adaptive testing (CAT), Cancer, Depression, EORTC CAT Core, Psychometric validation

## Abstract

**Purpose:**

The European Organisation for Research and Treatment of Cancer’s computerized adaptive test instrument, the EORTC CAT Core, provides dynamic, individualised assessment across 14 health-related quality of life domains, including emotional functioning (EF), which encompasses anxiety and depression. When one of these symptoms is of primary interest, separate scores may better support clinical decision-making and increase measurement sensitivity. The aim here was to evaluate separate item banks for anxiety and depression as potential supplements to the EORTC CAT Core.

**Methods:**

Responses to 33 candidate EF items from 1,023 cancer patients were available from the original EF item bank project. Expert-based content classification was followed by psychometric analyses, including confirmatory factor analysis (CFA), item response theory (IRT) model calibration and evaluation, differential item functioning (DIF) analyses, and CAT simulations to assess measurement properties of the two item banks.

**Results:**

Experts allocated nine items to anxiety and 17 to depression while seven items were judged to assess something else. CFA supported unidimensionality for each item bank. All items demonstrated acceptable IRT model fit, and no DIF of practical relevance was identified. CAT simulations indicated greater measurement precision than fixed 1- and 2-item scales, with potential sample size savings of up to 30% for anxiety and 50% for depression.

**Conclusion:**

Well-functioning and psychometrically sound item banks can be formed for anxiety and depression, respectively. These findings support the use of separate anxiety and depression CATs as potential supplements to the EORTC CAT Core for more targeted assessment. Further studies are needed to confirm the content validity of the two item banks.

## Introduction

Cancer patients may experience psychological distress before, during, and after anti-cancer treatment. Such distress, including anxiety and depression, is an important aspect of mental health and health-related quality of life and can arise from the diagnosis itself, the treatment, fear of recurrence, or from uncertainty about the future. Consequently, anxiety and depression are commonly reported among people experiencing cancer (reported prevalences up to 56% (anxiety) and 66% (depression), respectively) [1–6].

Although there is no generally agreed upon definition of emotional functioning (EF), it is often regarded as a higher-order construct closely related to psychological distress, encompassing aspects of mood and affect, such as anxiety and depressive symptoms [7]. EF can be assessed using both clinician-administered tools and patient-reported outcome measures (PROMs), the latter offering a direct insight into patients’ own experiences [7–11]. The European Organisation for Research and Treatment of Cancer (EORTC) Quality of Life Group’s (QLG’s) widely used core questionnaire, the QLQ-C30, includes a 4-item EF scale [12, 13]. This scale includes two items assessing anxiety and one on depression (the fourth is an indicator of general distress/non-specific negative affect). To allow more flexible and precise assessment, the QLQ-C30 EF scale was expanded into a 24-item item bank [14, 15]. An item bank is a collection or pool of calibrated items measuring the same underlying construct on a common metric using item response theory (IRT) [16, 17]. This enables different subsets of items to be administered while still yielding directly comparable scores. The EF item bank was designed for computerised adaptive testing (CAT) and is part of the EORTC CAT Core [18, 19].

CAT is an adaptive assessment method that dynamically selects items based on previous responses. It enables the selection of items that are most informative for the individual respondent, thereby increasing measurement precision while reducing the number of items needed to obtain reliable scores. Importantly, CAT preserves direct comparability across individuals and populations through the use of a common measurement metric [20, 21].

The EORTC CAT Core EF item bank was developed to offer these benefits while retaining conceptual alignment with the QLQ-C30 EF scale [14, 15]. Within this item bank, anxiety and depression were conceptualized as related aspects of a single unidimensional emotional functioning construct. The psychometric evaluations supported the use of such a unidimensional EF measure [15]. Nevertheless, although related, anxiety and depression represent conceptually and clinically distinct constructs, each with unique symptom profiles, underlying mechanisms, and potential implications for treatment [22, 23]. Hence, separate assessment of anxiety and depression is often desirable in both research and clinical practice. The question addressed in the present study is not whether or when anxiety and depression should be assessed separately, but whether the existing pool of EORTC EF items can support psychometrically sound item banks for separate assessment of the two constructs. If clinically validated, such item banks could provide more focused and responsive assessment of the construct being assessed. They may also help identify patients who could benefit from more targeted psychosocial interventions.

The aim of the present study was to investigate whether psychometrically sound separate item banks for anxiety and depression could be derived from the existing pool of EF items and hence potentially be added as supplements to the EORTC CAT Core instrument for more targeted assessment of the two constructs.

## Methods

### Emotional functioning items

The EORTC CAT Core includes 14 CAT item banks for the five functional and nine symptom domains of the QLQ-C30 questionnaire. The EF CAT item bank consists of 24 items and includes the four EF items of the QLQ-C30 [15]. All EORTC CAT Core measures use a T-score metric, scaled so that the European general population has a mean of 50 and a standard deviation of 10 for all domains [24]. In the current study, however, we applied the standard a priori N(0,1) metric for the latent trait distributions of anxiety and depression. These may later be transformed into T-scores.

During the development of the EF item bank, 33 distress candidate items were available for the final phase IV psychometric evaluation. Of these, nine items were discarded because they did not fit well to a 1-factor EF model [15]. These items were not discarded because of irrelevant content or the like, but mainly because the 1-factor structure favoured items tapping into several EF subdomains. Hence, all 33 items were candidates for the anxiety and depression item banks and were initially included here (item texts are provided in Table 1). All candidate items apply a 4-point ordinal response scale ranging from ‘not at all’ to ‘very much’ and a one week recall period [14].

**Table 1:** The 33 emotional functioning candidate items being evaluated

Item	Item text	Anxiety		Depression				
		Slope	Location	Slope		Location	Infit	Outfit
1^a^	Have you felt furious?							
2	Have you felt anxious?	2.25	0.77				0.87	0.74
3^b^	Did you feel tense?	2.22	0.83				0.87	0.75
4^b^	Have you felt helpless?			1.99		1.28	1.02	0.73
5^b^	Have you felt panic?	2.17	1.47				0.94	0.69
6^b^	Have you lost interest in things…?			1.19		1.34	1.01	0.90
7^b^	Have you felt vulnerable?			1.67		1.20	0.99	0.84
8^a,b^	Have you felt frustrated?							
9^b^	Have you felt worthless?			2.36		1.50	0.97	0.63
10	Have you felt that your life was meaningless?			2.26		1.66	0.94	0.75
11^a^	Have you felt angry?							
12^b^	Have you felt discouraged?			2.39		1.34	0.98	0.71
13^a,b^	Have you had emotional outbursts?							
14^b^	Have you felt that nothing could cheer you up?			3.24		1.50	0.98	0.58
15^b^	Have you felt afraid?	2.35	1.15				0.88	0.69
16^b^	Have you felt that pleasure has gone from your life?			2.16		1.30	0.96	0.73
17^b^	Have you had difficulty relaxing?	1.52	1.06				0.96	0.81
18^b^	Have you lost interest in your appearance?			1.34		1.92	1.02	0.88
19	Have you felt restless?	1.45	1.11				0.94	0.85
20^b^	Have you felt miserable?			3.04		1.12	0.93	0.64
21^a^	Have you been bad-tempered?							
22^b^	Did you feel depressed?			2.25		1.13	0.96	0.76
23^a,b^	Did you feel irritable?							
24^b^	Have you felt useless?			2.60		1.43	0.99	0.72
25^b^	Did you worry?	2.31	0.67				0.86	0.76
26^b^	Have you felt desperate?			2.40		1.65	1.04	0.65
27	Have you found it difficult to stop or control your worrying?	2.03	1.17				0.93	0.77
28^a^	Have you felt impatient?							
29^b^	Have you been afraid of losing control?	1.34	1.77				1.04	0.83
30^b^	Have you felt sad?			2.38		0.92	0.93	0.76
31^b^	Have you felt like giving up?			2.40		1.63	1.01	0.53
32^b^	Have you felt that you have nothing to look forward to?			2.43		1.49	0.95	0.64
33	Have you felt that life isn’t worthwhile?			2.10		1.81	0.92	0.69

^a^The experts judged the item assess something else than anxiety and depression, and hence, the item was not included in the psychometric validation

^b^The item is part of the EORTC CAT Core emotional functioning bank

### Expert-based content classification

Although an initial content classification of EF items was available from the original item bank development, a more structured consensus-based procedure was applied in the present study to ensure a formal allocation of items to anxiety and depression domains [14]. Four experts in the fields of psychology, oncology, and PROM development independently evaluated the conceptual content of the 33 items. For each item, the experts indicated whether it primarily assessed depression, anxiety, or another aspect of EF. If an item was judged to tap into additional domains beyond the primary classification, these were recorded as secondary classifications. For example, an item could be classified as primarily assessing depression but also assessing anxiety. An item was assigned to a domain if all experts agreed that the item assessed that domain, and at least half classified it as the primary domain. Items not meeting this criterion were discussed within the expert group until a consensus classification was reached. Items not assigned to either depression or anxiety were excluded from the following analyses.

### Psychometric validation

The psychometric validations were conducted separately for the two subsets of items classified by the experts as ‘anxiety’ and ‘depression’ items, respectively. The validation followed the procedure applied for the development of the EORTC CAT Core memory and attention item banks; further details can be found elsewhere [25]. All analyses were conducted using SAS Enterprise Guide version 8.4 except the factor analyses, which were conducted using CBID (https://biostats-shinyr.kumc.edu/CBID/).

#### Sample

The present study is based on secondary analyses of data collected between February and December 2011 for the psychometric evaluation of the EORTC CAT Core EF item bank. Patients were invited either by mail or during clinic visits to the department. Eligibility criteria included a verified cancer diagnosis, age ≥ 18 years, and sufficient physical and mental capacity to complete the questionnaire. The dataset consisted of responses from 1,023 cancer patients coming from Austria, Denmark, Italy, and the UK, with a mean age of 62 and 53% being women. Further details on the dataset are provided elsewhere [15].

#### Structural validation (confirmatory factor analysis)

The structural validity of the depression and anxiety item sets-i.e., their dimensionality-was assessed using confirmatory factor analysis (CFA) for ordinal data, assuming a 1-factor model for each domain [26]. That is, CFA was used as an assessment of essential unidimensionality to support subsequent IRT calibration rather than to identify the optimal latent structure. The 1-factor models were assessed using the following criteria for good/acceptable fit: the Tucker-Lewis Index (TLI) and the comparative fit index (CFI) each > 0.95, the root mean square error of approximation (RMSEA) < 0.08, and the standardized root mean square residual (SRMR) < 0.05 [27, 28].

#### Item response theory (IRT) model calibration and evaluation

First, the assumption of monotonicity, i.e., monotonic item response functions, was examined [29]. Next, a generalized partial credit model (GPCM) was fitted separately to the anxiety and depression item sets, consistent with the approach used for all EORTC CAT Core item banks [16, 18]. That is, the items allocated to each construct were recalibrated to a new GPCM with item parameters reflecting assessment of anxiety and depression, respectively. After initial model fitting, we evaluated potential local dependence-items being more strongly related than explained by the underlying trait alone-which may result in inflated slope parameter estimates [30]. Using a combination of Yen’s Q_3_ residual correlation statistic [31, 32] and inspection of item correlations and item slope estimates, we identified items showing indications of potentially problematic local dependence, i.e., potentially inflated slope estimates resulting from high (residual) correlations between items. To evaluate the possible impact of local dependence, items with high (residual) correlations with one or more other items and particularly high slope estimates were temporarily calibrated in reduced models excluding the highly correlated item(s). The resulting slope estimates were then compared with those obtained from the full calibration model. If a lower slope estimate was observed in the reduced model, the item slope parameter was fixed to this lower estimate and the item subsequently reintroduced into the full calibration model [25].

Item fit was evaluated examining infit and outfit statistics with high values (> 1.3) of particular concern as they may reflect underfit/poor model-data fit [15, 33], as well as plots of means of standardised item residuals with 95% confidence intervals (CIs) across levels of the domain score.

Differential item functioning (DIF) was examined using ordinal logistic regression across age, sex, cancer site and stage, treatment, country, education, cohabitation, and work status using the IRT domain scores as the matching criteria [34, 35]. Ordinal logistic regression allows for evaluation of both uniform and non-uniform DIF within a single modelling framework and has been found to work well for DIF detection [36, 37]. Given the large sample and multiple testing, DIF was considered statistically significant at *p* < 0.001, and only significant differences with a meaningful impact on domain scores were considered practically relevant. This was evaluated by comparing scores from the standard model with those from DIF-adjusted models, with differences exceeding the median standard error of the standard model scores flagged as potentially important [38, 39].

#### Evaluation of measurement properties of the item banks

The item information function is an estimate of the measurement precision of the item at each score level, with higher information corresponding to greater precision and smaller standard errors (SE = 1/(square root(information))). The sum of the item information functions across all items in a bank reflects the total measurement precision of the item bank [40]. The reliability of an item bank across the score continuum can be estimated from the information function by reliability = 1–1/information = 1−SE^2^ [40]. The total information function for each bank was evaluated and plotted across the score continuum. As an (arbitrary) criterion for high measurement precision, we defined the score range for which total information exceeded 10, corresponding to a reliability above 0.90.

For each domain, CAT performance was evaluated using simulations based on both observed and Monte Carlo-simulated data, covering fixed-length CATs of all lengths from one to all items in the bank. All anxiety CATs were initiated with the QLQ-C30 item about worry (item 25) while the depression CATs were initiated with item 22, the QLQ-C30 ‘depressed’ item. The resulting CAT-based domain scores were compared to scores based on all items of the domain bank. The correlation, median difference, and the percent differences <½ SD between scores were calculated. In line with the evaluations of the existing EORTC CAT Core item banks, including the EF item bank [15], the relative validity (RV) and the relative sample size requirement of the CAT-scores were assessed through known-groups comparisons against the corresponding QLQ-C30 domain item(s). Since the EORTC CAT was intended as an ‘improved version’ of the QLQ-C30, the original questionnaire was used as comparator or ‘benchmark’ for EORTC CAT performance. Therefore, the anxiety CAT-scores were compared against the sum score of the two QLQ-C30 anxiety items (item 3 and item 25) and depression CATs against item 22. Observed data comparisons included disease stage (I/II vs. III/IV), work status (working vs. not), treatment status (on treatment vs. not), and the QLQ-C30 sum scores for the 14 other domains dichotomised in scores < 33 vs. ≥33. As a summary, the median RV and relative sample size requirement were calculated and plotted for the varying length CATs following a previously presented approach [41]. For the simulated data, 1000 simulations were run per domain. In each simulation, two groups of random size between 50 and 250 were generated with random but known differences in true domain scores (corresponding to effect sizes ranging from 0.2 to 1.2).

## Results

### Expert-based content classification

The experts’ content classifications were in agreement for 30 of the 33 items (91%). The three remaining items (items 2, 11, and 20) were discussed until consensus agreements were reached. This resulted in nine items allocated to anxiety and 17 items to depression. The remaining seven items were judged to assess something other than anxiety and depression (Table 1) and were not included in the psychometric analyses.

The present classification was highly consistent with the original EF item bank development classification, with only three items differing in allocation (one from depression to anxiety, one from depression to general distress, and one from general distress to anxiety).

### Psychometric validation

The item subsets allocated by the experts to anxiety and depression, respectively, were analysed separately. All item response categories ((9 + 17)*4 = 104 in total) were endorsed by at least 15 respondents, with all but eight categories endorsed by at least 25 respondents allowing for including all items in the CFA and IRT models.

#### Structural validation (confirmatory factor analysis)

The CFA for the 17 depression items indicated good fit to a 1-factor model: CFI, TLI = 0.98, RMSEA = 0.07, and SRMR = 0.04. For the anxiety items, three of the four fit indices indicated good fit (CFI = 0.97, TLI = 0.96, SRMR = 0.047) while RMSEA = 0.12 exceeded our pre-specified criterion for good fit. However, it has been shown that highly correlated items may lead to elevated/poor values of RMSEA (RMSEA > 0.10) while the CFI indicates good fit (CFI > 0.95) [42]. Our primary aim at this step was to assess the unidimensionality of the item sets, and high inter-item correlations are not a concern in this context; such correlations may violate other IRT model assumptions, which are evaluated in the next analytical steps. Therefore, we concluded that both item sets were sufficiently unidimensional, and we retained all items for the subsequent analyses.

#### Item response theory (IRT) model calibration and evaluation

##### Anxiety

Seven (6%) of the checks for monotonicity indicated possible deviations from monotonicity. All were minor (< 3% of the score range) and non-significant (*p* > 0.35). Hence, no credible violations of monotonicity were detected and all nine items were included in the IRT model calibration.

Three of 36 correlations between standardised item residuals (Yen’s Q_3_ statistic) were above 0.25 indicating potential local item dependence: corr(item 2, item 19) = 0.26, corr(item 2, item 27) = 0.30, corr(item 17, item 19) = 0.25. As these were just above 0.25, they were not strong indications of local dependence. We tried removing each of the items from the model. This had only minor impact on the calibration and fit of the remaining items and nothing indicated generally improved fit if any of these items were removed (details omitted). Further, no item pairs showed indications of substantial overlap in content (all correlations < 0.70) and there were no indications of inflated item slopes either. Therefore, we included all items in the model, calibrating all items in the full model. This resulted in slope and location parameters as shown in Table 1.

Inspections of threshold parameters indicated ordered category thresholds for all items and category characteristic curves indicating appropriate ordinal functioning of all item response options (details omitted). Infit statistics ranged 0.86–1.04, and outfit statistics ranged 0.69–0.85, all well below our threshold for underfitting items (Table 1). Note that the infit and outfit values less than 1.0 indicated less variability than expected, which may suggest some redundancy among the items.

**Fig. 1:**
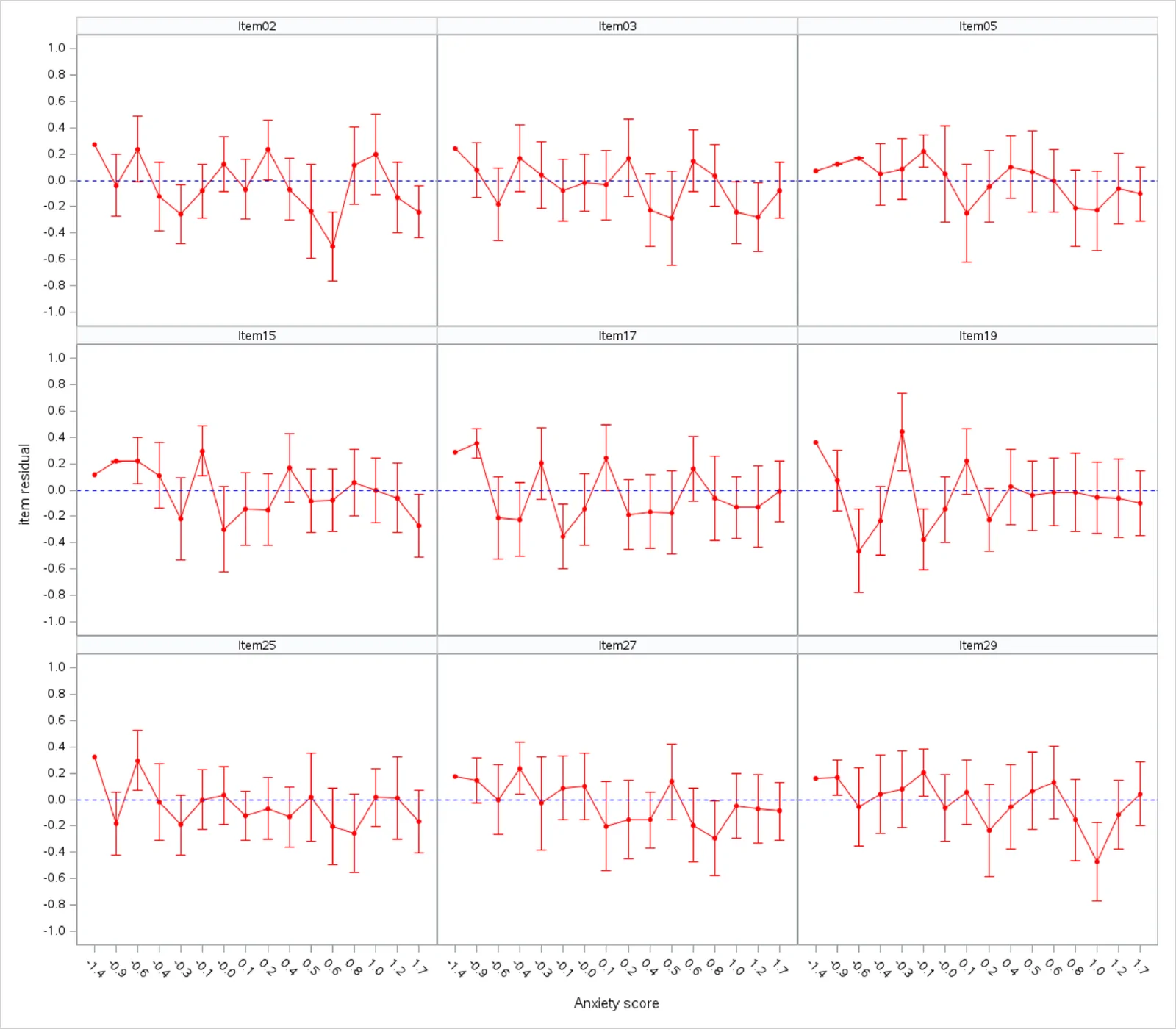
Means of standardised item residuals with 95% CI across anxiety scores

Plots of means of standardised item residuals with 95% CI are presented in Fig. 1. All residual means were within ± 0.5 (±½SD) and approximately 95% were within ± 0.3. Most of the confidence intervals crossed zero and were fully within ± 0.5. There did not appear to be any systematic patterns in the deviations. The mean residuals seemed to vary randomly across anxiety scores. Taken together, all items seemed to fit the model sufficiently.

No tests for non-uniform DIF were significant. Of the 81 tests for uniform DIF, six were significant, five when comparing countries (items 2, 3, 17, 27, 29) and one when comparing cancer sites (item 15). Comparing domain scores based on the standard model to scores based on a DIF-adjusted model, all differences were less than the median standard error (all differences < 0.3, 95% <0.1).

##### Depression

Of the checks for monotonicity, 26 (12%) indicated possible deviations from monotonicity. However, as all were minor (one = 3.8%, the rest < 2% of the score range) and non-significant (*p* > 0.15), these did not seem to reflect credible, relevant violations of monotonicity. Therefore, all items were included in the IRT model calibration.

All pairwise correlations between standardised item residuals were less than 0.25, i.e., these residual correlations did not indicate local item dependence. However, inspection of item correlations showed high correlations (ρ > 0.8) among items 9, 10, 24, and 31–33 indicating substantial overlap in content and hence, risk of inflated slope estimates. To reduce this risk, the slopes for these items were calibrated separately, for each item excluding highly correlated items. The resulting slope estimates were then fixed and the full model was calibrated. Table 1 present the resulting parameter estimates.

As for anxiety, threshold parameters and category characteristic curves indicating appropriate ordinal functioning of the item response options (details omitted). Infit statistics for the final model ranged from 0.92 to 1.04, and outfit statistics ranged 0.53–0.90, all well below our threshold for underfitting. In particular, the low outfit values-seven were below 0.7-indicated less variability than expected by the model.

**Fig. 2:**
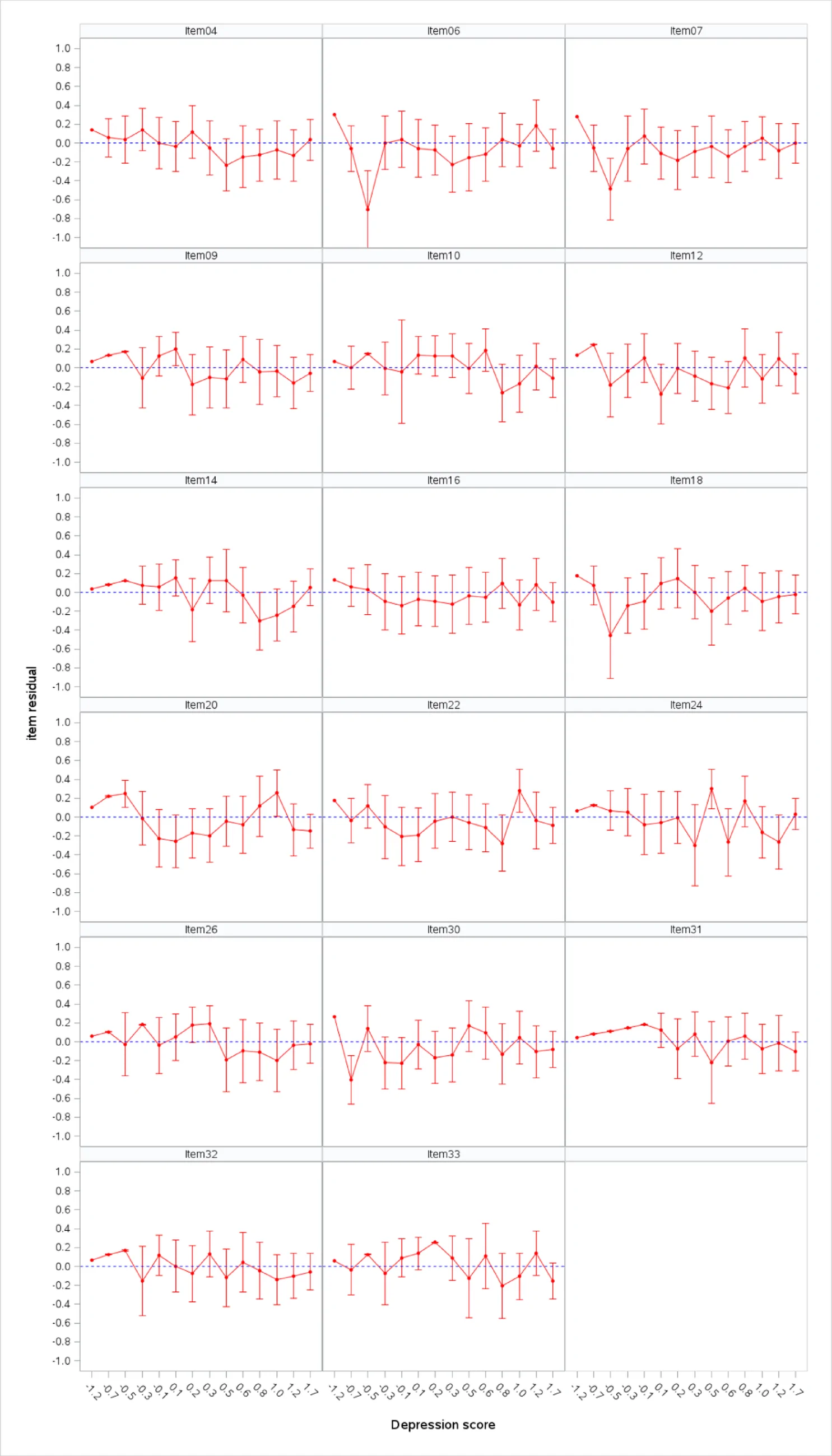
Means of standardised item residuals with 95% CI across depression scores

Figure 2 shows mean item residuals with 95% CI for the 17 candidate items. All residual means except one (item 6) were within ± 0.5 and all but five (2% of all residuals) were also within ± 0.3. The majority of the confidence intervals crossed zero and were within ± 0.5. The mean residuals seemed to vary randomly across depression scores without any systematic patterns to the deviations. Taken together, all items appeared to fit the model sufficiently.

There were no indications of non-uniform DIF. Eleven of the 153 tests for uniform DIF were significant: items 10, 12, 16, 18, 20, 22, 26, 31 showed DIF when comparing countries, item 4 when comparing stages, item 7 comparing levels of education and item 33 comparing sexes. However, none of these resulted in score differences between the standard model and the DIF-adjusted model exceeding the median standard error (all differences < 0.1).

#### Evaluation of measurement properties of the item banks

Full-bank scores for the anxiety and depression item banks, respectively, correlated 0.78 confirming they are assessing closely related but separate constructs and that the item banks may each provide unique information.

**Fig. 3:**
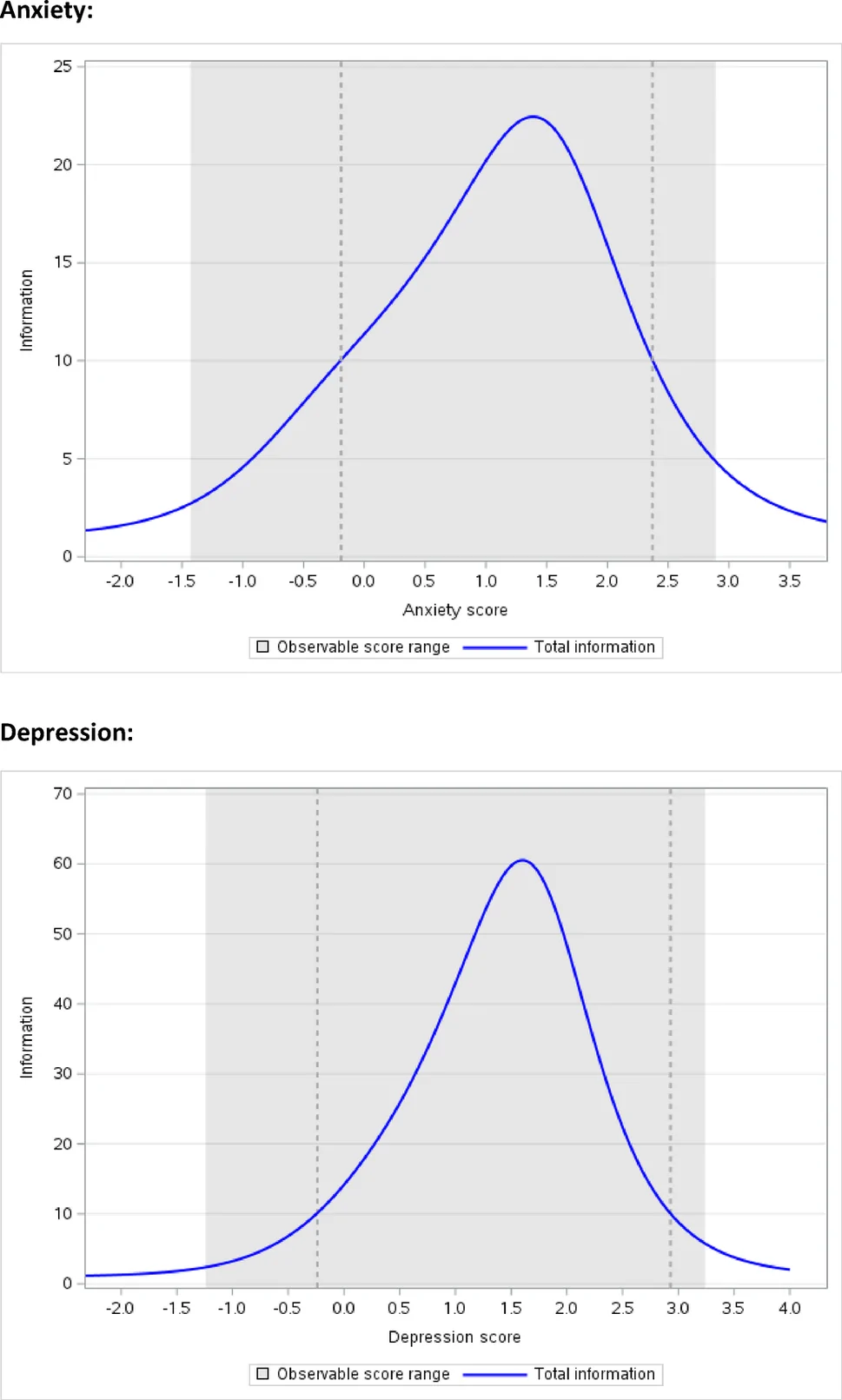
Total information functions for the anxiety and depression item banks, respectively. Note: For scores within the vertical dashed lines the item banks provided total information >10, corresponding to reliability >0.90.

Figure 3 shows the total information of the anxiety and depression item banks, respectively. For anxiety the total information was > 10, corresponding to reliability > 0.90, for scores ranging − 0.2 to 2.4 = 2.6 SD. For depression the reliability was > 0.90 for scores − 0.2 to 2.9 = 3.1 SD. For both item banks the information functions showed pronounced peaks, both centred around scores of 1.5, i.e., for those often reporting ‘quite a bit’ anxiety/depression symptoms.

**Fig. 4:**
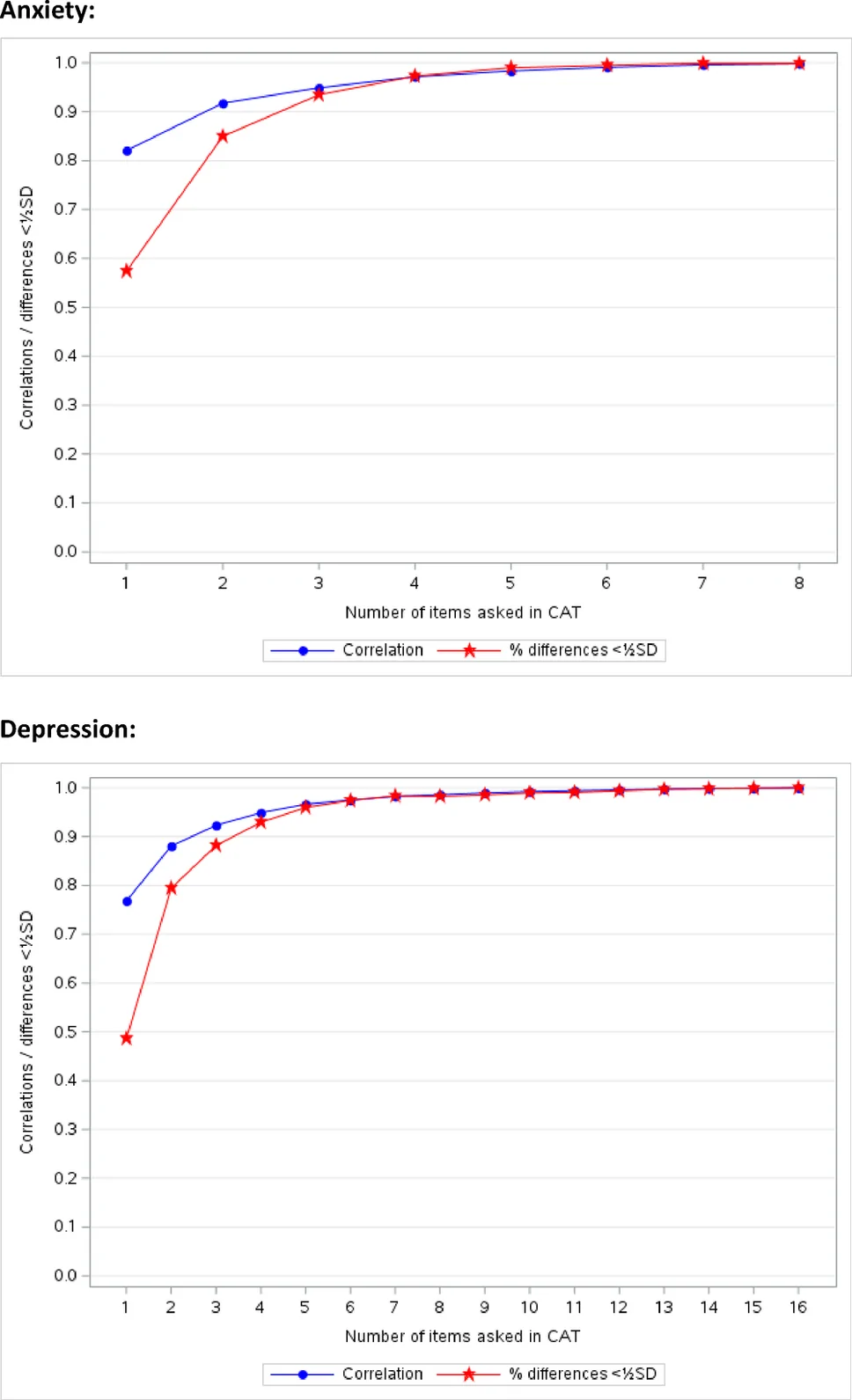
Correlations and percent differences <½SD for fixed-length CATs compared with scores from the full item banks for anxiety and depression, respectively

For both anxiety and depression, the median differences between CAT scores and full-bank scores were close to zero (< 0.1) for all CAT lengths (details not shown), indicating no systematic bias in the CAT scores. Correlations and percent score differences < 0.5 (½SD) between CAT scores and full-bank scores are shown in Fig. 4. Asking two or more anxiety items yielded correlations > 0.90, and asking three or more items resulted in more than 90% of CAT scores being within 0.5 points of the scores based on all nine anxiety items. For the depression bank, three items were needed to reach correlations > 0.90 with scores based on the full item bank, and four items were needed for more than 90% of CAT scores to be within 0.5 points of the scores based on all 17 depression items.

**Fig. 5:**
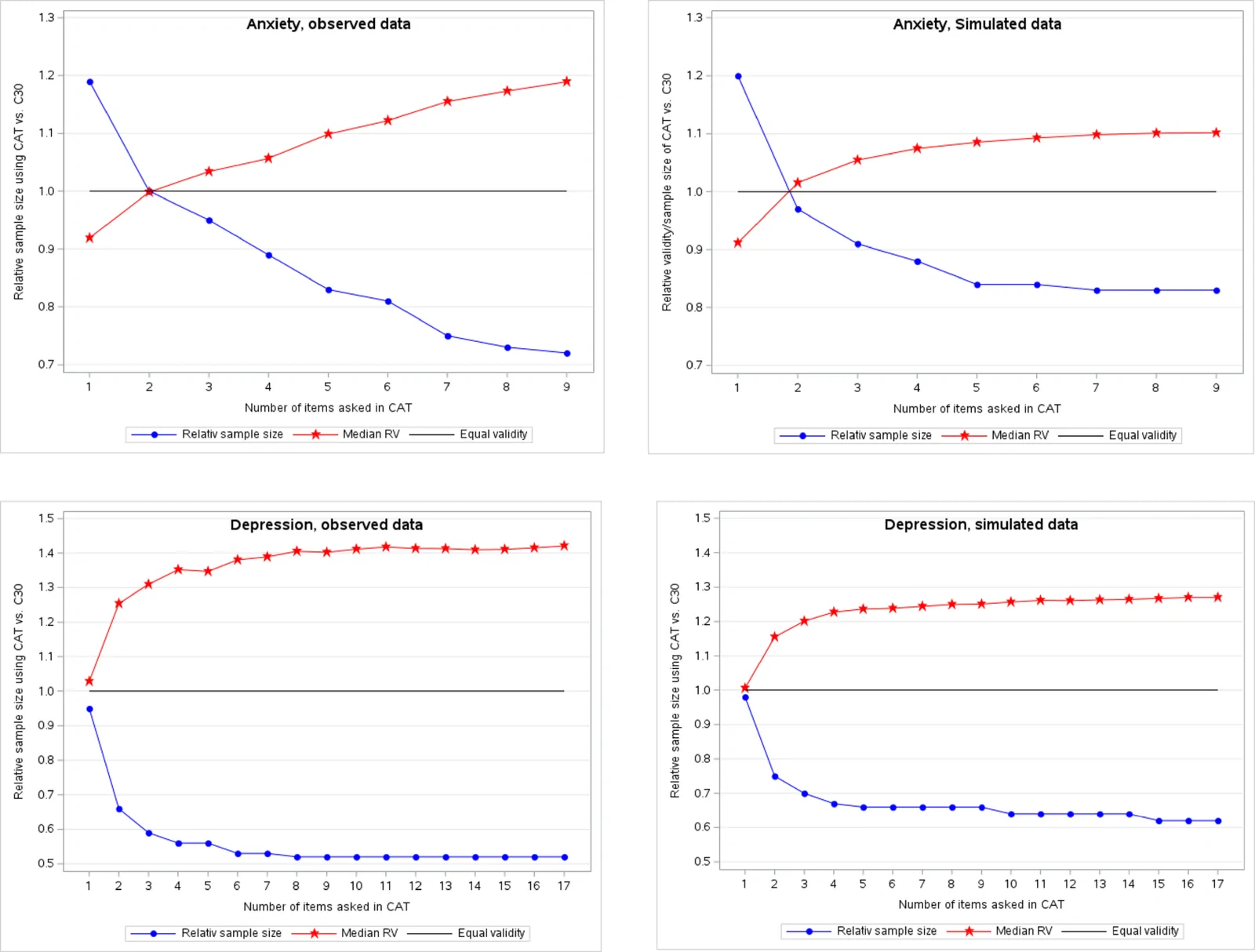
Relative validity (RV) and relative sample size requirement for fixed-length CATs compared to using the QLQ-C30 item(s) for anxiety and depression, respectively.

Figure 5 shows the median relative validity (RV) and the corresponding estimated relative sample size requirements for CATs of varying lengths compared with the QLQ-C30 item(s) sum scored. For anxiety CATs, compared with the sum score based on the two anxiety QLQ-C30 items (items 3 and 25), asking only one item provided-unsurprisingly-lower precision. In contrast, observed and simulated data showed higher precision (i.e., lower estimated sample size requirements) when asking two or more items. In the observed data, sample size requirements decreased steadily with additional items, reaching a maximum saving when using all nine items, which required approximately 30% fewer participants to achieve the same power as the QLQ-C30 scale. The evaluations on simulated data indicated that asking more than five items had minimal impact on precision; CATs asking five or more items all yielded estimated sample size savings just above 15%.

For depression, the evaluations indicated substantial sample size savings when using CATs with two or more items compared with the QLQ-C30 depression item (scored using standard scoring). In general, the observed data showed slightly greater savings than the simulated data. Taken together, the evaluations suggested that compared to asking one item, CATs asking two items may require approximately 25–35% fewer participants than the QLQ-C30 item; CATs asking five items may yield savings of about 35–45%; and asking all 17 depression items may yield sample size savings of roughly 40–50%.

## Discussion

This study provides a comprehensive psychometric evaluation of separate anxiety and depression item banks derived from the EORTC CAT Core emotional functioning item bank. This included items discarded from the original emotional functioning bank because they did not fit the unidimensional construct, but which were reconsidered here given the narrower anxiety and depression constructs. We aimed to determine whether each construct could be measured reliably and efficiently using separate item banks, and whether CAT administrations could yield precise, unbiased scores comparable to full-bank administration. Overall, our findings supported the hypothesized unidimensional constructs and indicated that both item banks have strong psychometric properties, excellent measurement precision across relevant score ranges, and substantial efficiency gains compared to the QLQ-C30 anxiety and depression counterparts.

The initial expert content classification confirmed the conceptual distinctness of the two domains, with high agreement among experts (91%). The allocation of nine items to anxiety and 17 items to depression allows for the CAT assessment of both domains. Confirmatory factor analyses indicated unidimensionality for both banks, supporting the conceptual classification conducted by the experts. This allowed all items to be retained for IRT calibration. Standardized residual analyses showed no systematic patterns of item misfit, and infit and outfit statistics met our predefined fit criteria for all items, although several outfit statistics, particularly for depression, were low suggesting some redundancy in content across items. The outfit statistic is sensitive to outliers, meaning it is particularly sensitive to unexpected responses when the individual’s score level and the item’s difficulty do not match. In our case the main reason for the low outfit values was likely floor effects: those with the lowest obtainable anxiety/depression score responded ‘not at all’ more than the models expected. When removing those with the minimum score, all outfits exceeded 0.7, confirming this assumption. Such ‘redundancy’ is not inherently problematic, but it reflects that the item banks may lack relevant items for those with very low levels of anxiety/depression (those reporting ‘not at all’ symptoms on more than half of the items). This was also confirmed by the plots of information functions. From a clinical perspective, lower precision for absent to very low symptom levels should have little impact, as the aim is to identify patients experiencing mental health problems. Nevertheless, although the item banks demonstrated acceptable psychometric properties, particularly the anxiety item bank may benefit from additional item development to broaden coverage and further improve measurement precision across the full score continuum.

DIF analyses identified a limited number of statistically significant DIF effects, mainly by country. However, none of these seemed to have any practical impact on measurement invariance. This supports the application of both banks across different clinical and cultural groups.

Both banks demonstrated reliability > 0.90-here interpret as high measurement precision-across acceptable score ranges: 2.6 SD for anxiety and 3.1 SD for depression. Information function reached its maximum around 1.5, corresponding to individuals reporting on average ‘quite a bit’ of symptoms. That is, the item banks are highly informative and thus well-suited for detecting and discriminating among patients with moderate to high symptom levels. Precise assessment among these patients may be particularly relevant in clinical use and intervention studies.

Simulations of CAT assessments based on both observed and simulated data demonstrated that short CATs could reproduce full-bank scores with high accuracy and precision. For example, correlations > 0.90 with full-bank scores were achieved for CATs with ≥ 2 anxiety items and ≥ 3 depression items, respectively. Relative validity analyses further highlighted the efficiency of CAT administration. Compared to short sum scales of QLQ-C30 items, the simulations indicated clear sample size savings, and therefore potentially reduced study costs, using the CATs without compromising statistical power. For example, it was estimated that depression CATs asking just two questions may reduce sample size requirements by 25–35% compared to using the QLQ-C30 depression item. As anxiety and depression have not previously been defined as QLQ-C30 scales, these comparisons are necessarily hypothetical; still, the results indicate that, when these constructs are targeted, brief CATs offer a more efficient alternative.

The simulations yielded slightly higher RVs for CATs when based on observed data. It remains unclear which approach-simulations or observed data-is closer to the “truth” when evaluating sample size requirements. Observed data reflect real response patterns but may involve suboptimal group contrasts, whereas simulated data rely on assumptions that may not fully mirror actual behaviour but are based on true, known group differences. Both approaches therefore offer complementary insights, and it may be beneficial to triangulate the observed and simulated results, e.g., by calculating a simple average.

Our findings align with a prior study showing the feasibility and efficiency of domain-specific CATs for memory and attention developed based on the EORTC CAT Core cognitive functioning item bank [25]. Together, these studies confirm that well-functioning domain-specific CAT item banks may be formed based on existing banks for composite domains and thereby expand the flexibility and specificity of the EORTC CAT Core system. Consistent with their theoretical relatedness, anxiety and depression were strongly correlated (*r* = 0.78). However, the correlation was well below 1.0 (shared variance ≈ 61%), indicating substantial non-overlapping variance and supporting the relevance of separate item banks. The distinction between anxiety and depression as related but separable constructs is well established in clinical psychology and has been reflected in other PROMs such as the HADS and PROMIS and outcome standardisation initiatives (e.g., ICHOM and COMET) [8, 43–45]. Hence, the present work is consistent with a broader tradition of domain-specific measurement of anxiety and depression.

Major strengths of this study include the large, international sample and the use of both observed and simulated data for CAT performance evaluations. This dual approach enhances confidence in the robustness of the results. The analyses were conducted according to modern IRT standards, including rigorous assessment of unidimensionality, item fit, and DIF-another strength of the study.

Some limitations should be acknowledged. First, although expert reviews and psychometric evaluations supported the allocation of items to anxiety and depression, the present study did not include external validation against independent measures of anxiety and depression. Consequently, convergent and discriminant validity could not be formally evaluated. Hence, the content validity of the two item banks warrants further evaluation to ensure that the banks fully capture the intended symptoms. Although patient interviews were conducted during the original development of the emotional functioning item bank, these did not explore patient perspectives on distinguishing anxiety and depression as separate constructs. Future evaluations should ideally also include the patient perspective. Second, the possible redundancy (local dependence) among items and limited precision for low symptom levels indicate opportunities for further bank refinement, such as the removal or rewording of items with overlapping content and addition of items specifically relevant for low symptom levels. While we aimed to reduce inflation of slope estimates caused by local dependence, some residual local dependence may remain and could result in overestimation of total information when locally dependent items are administered together. In CAT administration, this can be mitigated by preventing locally dependent item pairs from being selected within the same assessment. Third, the efficiency gains observed in the relative validity analyses should be interpreted as relative to very brief measures (1–2 items) and not necessarily to an optimal fixed-length static form. Finally, while model comparisons between a 1-factor emotional functioning model and a 2-factor anxiety-depression model were not the focus of the present study, such analyses may be informative to further evaluate the relative performance of alternative representations of emotional functioning. Such evaluations are encouraged in future work.

## Conclusion

Separate anxiety and depression item banks derived from the EORTC CAT Core emotional functioning item bank demonstrated strong psychometric properties, high measurement precision, and potential efficiency gains compared with QLQ-C30 based scales. Evaluations indicated CAT administrations generally provided unbiased scores and may reduce sample size requirements up to 30% for anxiety and 50% for depression. These findings support the use of separate anxiety and depression measures. Although both item banks derive from the extensively validated EORTC emotional functioning scale, additional work is needed to confirm the content validity of the two item banks, including comparisons with established instruments. In particular, clinical validation against instruments used for clinical diagnoses of anxiety and depression are encouraged before clinical use.

## Data Availability

Data may be available from the EORTC upon reasonable request.
